# Molecular mechanism of unloading-mediated muscle atrophy and development of its countermeasures

**DOI:** 10.1186/ar3599

**Published:** 2012-02-09

**Authors:** Takeshi Nikawa

**Affiliations:** 1 Department of Nutritional Physiology, Institute of Health Biosciences, The University of Tokushima Graduate School, Japan

## 

**Figure 1:**
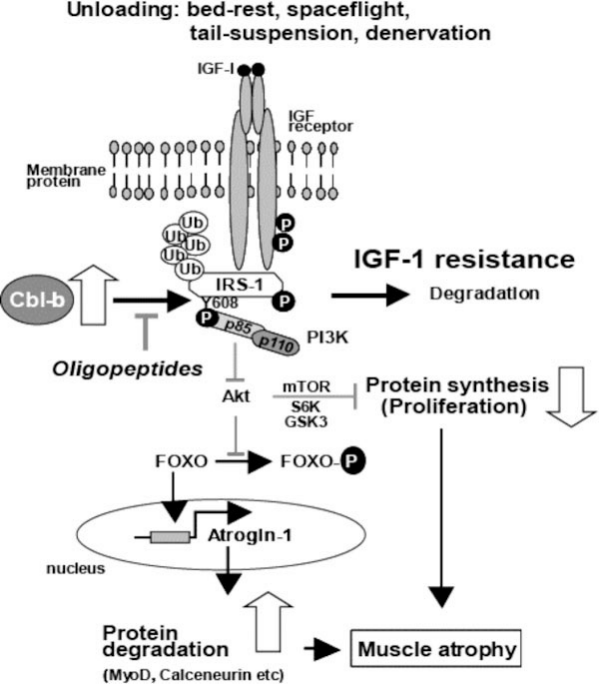
**Possible mechanism of unloading-mediated muscle atrophy**.

The ubiquitin ligase Cbl-b plays a major role in skeletal muscle atrophy induced by unloading [1]. The mechanism of Cbl-b-induced muscle atrophy is unique in that it does not appear to involve the degradation of structural components of the muscle, but rather it impairs muscular trophic signals in response to unloading conditions. Recent studies on the molecular mechanisms of muscle atrophy have focused on the role of IGF-1/PI3K/Akt-1 signaling cascade as a vital pathway in the regulation of the balance between hypertrophy and atrophy [2,3]. These studies indicate that under muscle wasting conditions, such as disuse, diabetes and fasting, decreased IGF-1/PI3K/Akt-1 signaling augments the expression of atrogin-1, resulting in muscle atrophy. However, these studies did not address the mechanisms of unloading-induced impairment of growth factor signaling. In the present study, we found that under both *in vitro *and *in vivo *experimental conditions, Cbl-b ubiquitinated and induced specific degradation of IRS-1, a key intermediate of skeletal muscle growth regulated by IGF-1/insulin and growth hormone, resulting in inactivation of Akt-1. Inactivation of Akt-1 led to upregulation of atrogin-1 through dephosphorylation (activation) of FOXO3, as well as reduced mitogen response, in skeletal muscle. Thus, activation of Cbl-b may be an important mechanism underlying the failure of atrophic muscle to respond to growth factor-based treatments such as IGF-1 (Figure 1).
